# ﻿The genus *Apterygothrips* Priesner (Thysanoptera, Phlaeothripinae, Haplothripini) from China, with one new species

**DOI:** 10.3897/zookeys.1112.85902

**Published:** 2022-07-12

**Authors:** Lihong Dang, Linpeng Zhao, Yanqiao Li, Gexia Qiao

**Affiliations:** 1 School of Bioscience and Engineering, Shaanxi University of Technology, Hanzhong, 723000, China Shaanxi University of Technology Hanzhong China; 2 Shaanxi Changqing National Nature Reserve, Changqing Jiayuan, No.176 Dongyi Huan Road, Hanzhong, Shaanxi 723000, China Shaanxi Changqing National Nature Reserve, Changqing Jiayuan Hanzhong China; 3 Key Laboratory of Zoological Systematics and Evolution, Institute of Zoology, Chinese Academy of Sciences, No.1 Beichen West Road, Chaoyang District, Beijing 100101, China Key Laboratory of Zoological Systematics and Evolution, Institute of Zoology, Chinese Academy of Sciences Beijing China; 4 College of Life Science, University of Chinese Academy of Sciences, No. 19, Yuquan Road, Shijingshan District, Beijing 100049, China University of Chinese Academy of Sciences Beijing China

**Keywords:** *Apterygothripsflavescens*, CO1, Haplothripini, key, new species

## Abstract

The genus *Apterygothrips* Priesner is a group of mainly wingless species in the tribe Haplothripini. The genus is diagnosed here based on the three species known from China, including *A.flavescens* Dang & Qiao **sp. nov.** collected from bases of grass in Tibet. An illustrated identification key to the three species from China is provided and the CO1 barcode sequence is given for the new species.

## ﻿Introduction

Species of *Apterygothrips* Priesner in the tribe Haplothripini are distributed worldwide and almost always wingless. Although there are now 39 species listed in this genus (ThripsWiki 2022), it has always been a weakly diagnosed group that is closely related to the huge genus *Haplothrips*. Species of *Apterygothrips* cannot be distinguished from wingless *Haplothrips* species, except for antennal segment IV bearing two or three sense cones and pelta trapezoidal or hemicircular. In *Haplothrips* species, antennal segment IV usually bears four sense cones and the pelta is triangular or with lateral lobes. Moreover, biological information about species of this genus is often unclear, with species likely to be phytophagous, fungus-feeding or predatory. Three species living on green plant tissues were described from Spain and Israel by zur Strassen (1966): *A.longiceps* zur Strassen on young shoots of *Ericaarborea*, *A.piceatus* zur Strassen in *Crataegus* flowers and *A.priesneri* zur Strassen on leaves of *Pinushalepensis*. In contrast, Mound and Wells (2015) recorded *A.sparsus* Mound & Walker as collected together with large populations of mites, suggesting that it may be predatory. Some species of the genus from the Asian region are known from dead leaves and branches (Bhatti and Mehra 1994; Bhatti 1997; Okajima 2006) and may possibly be fungus-feeding. The genus *Apterygothrips* remains complicated, and thrips workers simply placed species of Haplothripini with reduced wings and antennal segment IV with 2 or 3 sense cones in this poorly-defined genus. Four species of *Apterygothrips* are recorded from New Zealand, but each of these is known to produce macropterae although only in very low numbers (Mound and Walker 1986). The genus needs more evidence, such as combining morphological characters with molecular data, to confirm the relationship of its members as a monophyletic group.

At least eight species of *Apterygothrips* are listed from Asia (ThripsWiki 2022), only two of which were recorded from China prior to this study: *A.brunneicornus* Han and *A.haloxyli* Priesner (Han et al. 1991; Han 1997). As part of ongoing research on Haplothripini from China, we focused, in this study, on the wingless species. Three such species are recognized here, including the two named above and one new species. The new species is placed in the genus *Apterygothrips* because of the following major structures: slender body, wings absent, antennal segment III with one outer sense cone, antennal segment IV with two outer sense cones, head longer than wide, postocular setae well developed, pronotal notopleural sutures complete, basantra broader than long, pelta hemicircular and anal setae slightly shorter than tube. Because of these characters the new species could not be considered to belong to other associated genera such as *Haplothrips*, *Karnyothrips*, *Podothrips* and *Xylaplothrips*. The new species, *Apterygothripsflavescens* Dang & Qiao sp. nov., is described here and illustrated, and a key to the three *Apterygothrips* species from China is also provided.

## ﻿Materials and methods

The descriptions, photomicrograph images and drawings were produced from slide-mounted specimens with an Olympus BX53 and drawing tube. The abbreviations used for the pronotal setae are as follows: **am** – anteromarginal, aa – anteroangular, ml – midlateral, **epim** – epimeral, pa – posteroangular. The unit of measurement in this study is the micrometre. The genomic DNA of *Apterygothripsflavescens* Dang & Qiao sp. nov. was extracted from single specimens following the standard protocol of DNeasy kit (Qiagen, Hilden, Germany). The CO1 sequence was amplified using primers LCO1490 and HCO2198 (Mound et al. 2010). Sequence was assembled by Seqman II (DNAstar, Inc., Madison, WI, USA) and then aligned using Clustal W.

All specimens studied here are deposited in the School of Bioscience and Engineering, Shaanxi University of Technology (**SUT**), Hanzhong, China, and in the National Zoological Museum of China (**NZMC**), Institute of Zoology, Chinese Academy of Sciences, Beijing, China.

## ﻿Taxonomy

### ﻿ Apterygothrips

Taxon classificationAnimaliaThysanopteraPhlaeothripidae

Priesner

96345ECB-DA65-5DA6-8EB1-553DA4ECDA02

Apterygothrips Priesner, 1933: 153. Type species: Apterygothripshaloxyli Priesner, 1933.

#### Diagnosis.

Micropterous or apterous (rarely macropterous). Body uniformly brown or bicolored. Head longer than wide, eyes normal or small, ocelli small or absent, postocular setae well developed, pointed or capitate at apex; stylets about one third of head width apart, maxillary bridge present. Antennae eight-segmented, antennal segment III with one or two sense cones, IV with two or three. Pronotum usually smooth, with five pairs of major setae, sometimes am or (and) ml reduced, notopleural sutures complete; basantra present; mesopresternum boat-shaped or eroded medially; metathoracic sternopleural sutures absent. Fore tarsal tooth small or absent. Pelta trapezoidal or hemicircular; tergites II–VII with or without two pairs of weak, wing-retaining setae; tube shorter than head.

### ﻿Key to *Apterygothrips* species from China

**Table:** 

1	Body bicolored, with pterothorax yellow	***flavescens* Dang & Qiao, sp. nov.**
–	Body uniformly brown	**2**
2	Major setae pointed at apex; antennal segment IV with 1+2 sense cones; fore tarsal tooth absent	***brunneicornus* Han**
–	Major setae capitate at apex, or at least epimeral setae expanded; antennal segment IV with 1+1 sense cones; fore tarsal tooth present	***haloxyli* Priesner**

### ﻿ Apterygothrips brunneicornus

Taxon classificationAnimaliaThysanopteraPhlaeothripidae

Han

FBDFCD56-054B-52E1-88F4-9B4986DD13CE

Figs 1–5 , 21

Apterygothrips brunneicornus Han, 1991: 337.

#### Comments.

**Figures 1–5.**
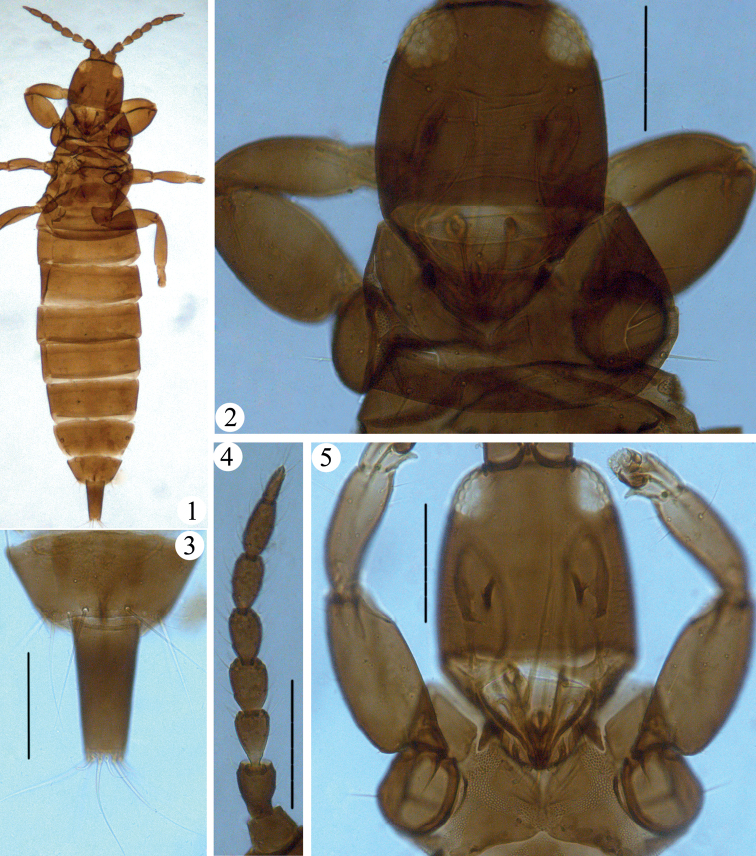
*Apterygothripsbrunneicornus*. Holotype female (**1–4**) **1** adult **2** head, pronotum and fore legs **3** tergites IX–X **4** antenna; paratype female (**5**) **5** paratype female: head, pronotum and fore legs. Scale bars: 100 μm.

This species was described by Han et al. (1991) from Sichuan Province and Tibet based on two females. In the present study, we checked the two type specimens. They were squashed (Fig. 1), but fortunately the major characters could be seen. *Apterygothripsbrunneicornus* is similar to *A.flavescens* Dang & Qiao sp. nov. in having major setae pointed (Figs 2, 3), and it comes from the same location: Tibet. It can be distinguished by having body uniformly brown (Fig. 1), antenna brown with segment III paler at base (Figs 1, 4), antennal segment IV with 1+2 sense cones, fore tarsal tooth present (Fig. 5), pronotum with five pairs of well-developed setae (Fig. 2) and by its pelta shape (Fig. 21).

### ﻿ Apterygothrips flavescens

Taxon classificationAnimaliaThysanopteraPhlaeothripidae

Dang & Qiao sp. nov.

B33D2AA1-B0B7-549A-8BD7-7911ED1E81C3

https://zoobank.org/7FB37E68-5A47-4448-AD7C-319BCE46BE3C

Figs 6–13 , 14–20

#### Material examined.

***Holotype*.** ♀ (SUT), China, Tibet, Lhasa City, Lhasa Nanshan Park, from base of grass, 03.viii.2019, L.H. Dang and L.P. Zhao. ***Paratypes*.** 1♀3♂ (SUT), same data as holotype.

#### Diagnosis.

**Figures 6–13.**
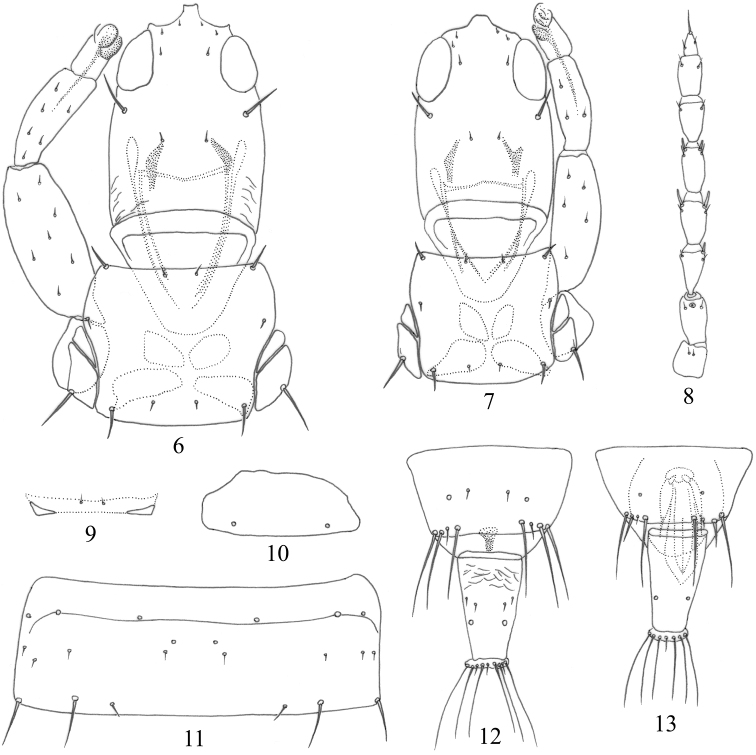
*Apterygothripsflavescens* Dang & Qiao sp. nov. **6** head, pronotum and fore leg, female **7** head, pronotum and fore leg, male **8** antenna **9** mesopresternum **10** pelta **11** abdominal tergite V **12** abdominal tergites IX–X, female **13** abdominal tergites IX–X, male.

**Figures 14–21.**
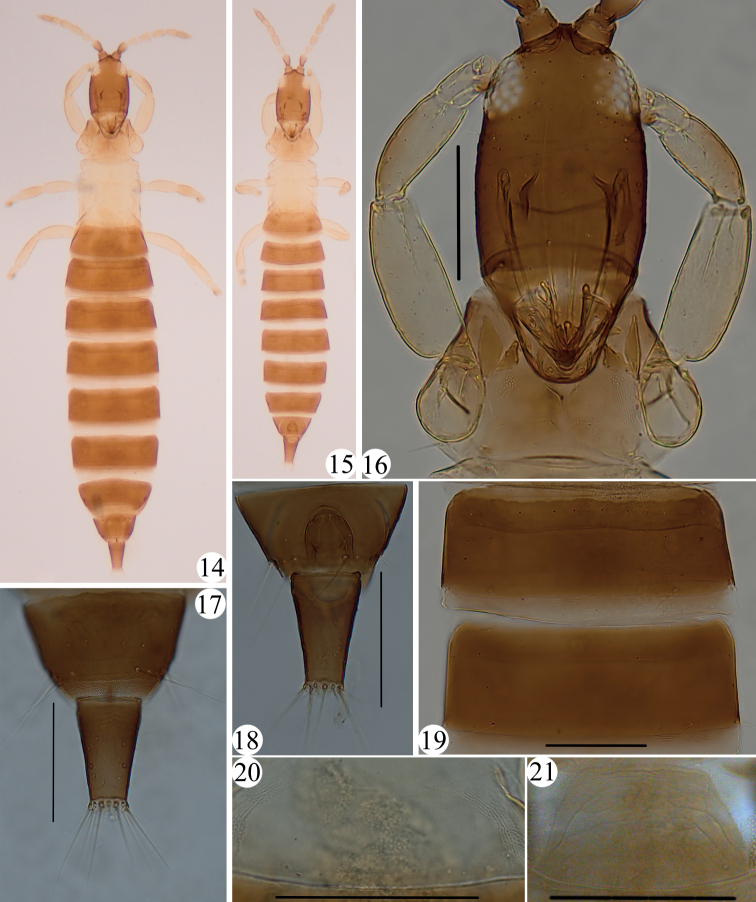
*Apterygothrips* spp. *A.flavescens* Dang & Qiao sp. nov. (**14–20**) **14** adult, female **15** adult, male **16** head, pronotum and fore legs **17** tergites IX–X, female **18** tergites IX–X, male **19** tergites V–VI **20** pelta; *A.brunneicornus* (**21**): **21** pelta. Scale bars: 100 μm.

Apterous, body bicolored, with pterothorax yellow (Figs 14, 15); major setae pointed at apex. Head longer than wide, postocular setae shorter than eye (Figs 6, 7); pronotal am and ml minute, other three pairs of major setae well developed (Figs 6, 7); mesopresternum reduced to two small, lateral triangular plates, completely eroded medially (Fig. 9); fore tarsal tooth absent (Figs 6, 7, 16); Pelta hemicircular (Figs 10, 20); tergites II–VII without wing-retaining setae (Figs 11, 19); tube shorter than head, anal setae slightly shorter than tube (Figs 12, 13, 17, 18).

#### Description.

***Holotype*. *Female aptera*.** Body bicolored (Fig. 14), head and abdominal segments II–X brown, meso-, metathorax and pelta pale yellow, prothorax brownish yellow, color between head and pterothorax. All legs yellow with outer margins brown. Antennal segments I–II and VIII brown, III yellow, IV–VII gradually yellow to brownish, VIII brown. Abdominal pelta yellow (Fig. 20), concolorous with pterothorax, segments II–X uniformly brown. Body setae hyaline.

***Head*.** Head 1.6 times as long as wide (Figs 6, 16); dorsal surface almost smooth; ocelli absent; eyes not prolonged ventrally, postocular setae well developed but shorter than eyes, pointed at apex (Figs 6, 16); cheeks weakly rounded. Mouth-cone rounded, maxillary stylets retracted into postocular setae, about half of head width apart at middle (Fig. 16). Antenna 8-segmented, sensoria small, III with 0+1, VI with 1+1, V with 1+1 (Fig. 8).

***Thorax*.** Pronotum almost smooth, notopleural sutures complete (Fig. 6); am and ml minute, other three pairs of major setae well developed, pointed at apex, epim the longest (Fig. 6); mesopresternum reduced to two small, lateral triangular plates, completely eroded medially (Fig. 9); metanotum smooth, metathoracic sternopleural sutures absent. All legs normal, without fore tarsal tooth (Figs 6, 16).

***Abdomen*.** Pelta smooth and hemicircular, with a pair of campaniform sensilla (Figs 10, 20); abdominal tergites II–VII without wing-retaining setae (Figs 11, 19); tergite IX setae developed, but all shorter than tube, pointed at apex (Fig. 12); tube about 0.5 times as long as head, 1.6 times as long as basal width, anal setae slightly shorter than tube (Fig. 17).

***Measurements*** (holotype female in microns). Body length 1580. Head length 175, width across eyes 110; eye length 50, postocular setae length 35. Antenna length 265, segments I–VIII length (width) 25(30), 35(25), 35(20), 35(25), 35(20), 30(20), 25(20) and 20(10), sensoria on segment III length 10. Pronotum length 130, width 155, length of pronotal setae: am 5, aa 15, ml 5, epim 30, pa 25. Pelta length 65, width 125; tergite IX posteromarginal setae S1–S3, 65, 80, 75; tube length 80, basal width 50, apical width 30; anal setae length 75.

***Male aptera***. Very similar to female (Fig. 15), fore legs without fore tarsal tooth (Fig. 7); abdominal tergite IX setae S2 short and stout (Figs 13, 18), sternites without a pore plate.

***Measurements*** (paratype male in microns). Body length 1225. Head length 160, width across eyes 105; eye length 50, postocular setae length 30. Antenna length 250, segments I–VIII length (width): 25(25), 35(20), 30(20), 30(20), 30(20), 30(20), 25(20) and 20(10), sensoria on segment III length 10. Pronotum length 100, width 125, length of pronotal setae: am 5, aa 15, ml 5, epim 20, pa 10. Pelta length 45, width 95; tergite IX posteromarginal setae S1–S3, 70, 20, 85; tube length 75, basal width 50, apical width 25; anal setae length 75.

#### CO1 sequence.

It includes 1536 bp with the GenBank accession number ON350971.

#### Etymology.

The Latin name “flavescens” refers to the yellow pterothorax of the new species.

#### Comments.

The new species, *A.flavescens* Dang & Qiao sp. nov., is similar to *A.bicolor* Johansen and *A.dempax* Bhatti & Ananthakrishnan in having a bicolored body. But it differs in having head and abdominal tergites II–X brown, pterothorax and pelta pale yellow, prothorax brownish yellow and intermediate in color between head and pterothorax, and major setae pointed at apex. In *A.bicolor* from Mexico, the head, prothorax, mesothorax and apical two thirds of the tube are brown, the metathorax, abdominal segments I–IX and the basal third of the tube are pale yellow, and the major setae are expanded at the apex (from original description, Johansen 1982). In *A.dempax* Bhatti & Ananthakrishnan from India, the body is almost yellow except for abdominal tergites IX–X or only the tube brown, and the major setae are expanded at the apex (from original description, Bhatti and Ananthakrishnan 1978).

### ﻿ Apterygothrips haloxyli

Taxon classificationAnimaliaThysanopteraPhlaeothripidae

Priesner

8D6E198C-CD0F-5E9D-B499-67DA7ABCFA1E

Apterygothrips haloxyli Priesner, 1933: 1.

#### Comments.

This species was described by Priesner (1933) from Egypt, based on specimens collected on fallen petals of *Haloxylonschweinfurthi*. It was first recorded from China (Ningxia Autonomous Region) by Han (1997). Here, one female and one male identified by Han and four females and one male from Inner Mongolia were studied. *Apterygothripshaloxyli* can be distinguished from the other Chinese species by having major setae capitate at apex.

## Supplementary Material

XML Treatment for Apterygothrips

XML Treatment for Apterygothrips brunneicornus

XML Treatment for Apterygothrips flavescens

XML Treatment for Apterygothrips haloxyli
